# Cigarette smoke exposure redirects *Staphylococcus aureus* to a virulence profile associated with persistent infection

**DOI:** 10.1038/s41598-019-47258-6

**Published:** 2019-07-25

**Authors:** Alicia Lacoma, Andrew M. Edwards, Bernadette C. Young, José Domínguez, Cristina Prat, Maisem Laabei

**Affiliations:** 1 Servei de Microbiologia, Hospital Universitari Germans Trias i Pujol, Institut d’Investigació Germans Trias i Pujol, Universitat Autònoma de Barcelona, Badalona, Spain; 2 CIBER Enfermedades Respiratorias, Badalona, Spain; 3 MRC Centre for Molecular Bacteriology and Infection, Imperial College London, London, United Kingdom; 4 Nuffield Department of Medicine, Experimental Medicine Division, University of Oxford, Oxford, United Kingdom; 5 Department of Biology and Biochemistry, University of Bath, Bath, United Kingdom

**Keywords:** Applied microbiology, Pathogens

## Abstract

Tobacco smoking represents the leading preventable cause of death worldwide. Smoking is a recognised risk factor for several pathologies and is detrimental to host immune surveillance and defence. However, the impact of smoking on microbial residents of the nasopharyngeal cavity, in contact with cigarette smoke (CS), is lacking. *Staphylococcus aureus* is a major human pathogen that colonises the human nasopharynx and causes a wide range of infections. We investigated the impact of CS on specific virulence phenotypes important in *S aureus* pathogenesis. We observed strain-dependent differences following exposure to CS, namely growth inhibition, augmented biofilm formation, increased invasion of, and persistence within, bronchial alveolar epithelial cells. Additionally, we confirm the critical role of a functional accessory gene regulator (Agr) system in mediating increased biofilm development and host cell invasion and persistence following CS exposure. Furthermore, CS exposure resulted in reduced toxin production. Importantly, exposure of *S aureus* to CS accelerated the frequency of mutations and resulted in a significant increase in gentamicin-resistant small colony variant (SCV) formation. Mutational analysis revealed that CS induced SCVs emerge via the SOS response DNA mutagenic repair system. Taken together, our results suggest that CS redirects certain *S aureus* strains to a virulence profile associated with persistence.

## Introduction

The tobacco epidemic is best portrayed in the approximately six million people that died as a result of cigarette smoking in 2010 alone^1^. Smoking is responsible for 90% of lung cancers^2^, is associated with 50% of cardiovascular diseases^3^ and is directly correlated with the development of chronic obstructive pulmonary disease^4^. Critically, smoking increases the risk for several infectious diseases^5,6^, most notably in the manifestation of invasive pneumococcal infection^7^. Recently, cigarette smoke (CS) condensate has been shown to significantly increase the biofilm forming capacity of *Streptococcus pneumoniae*, potentially augmenting pneumococcal persistence and resistance to antibiotics^8^. Smoking is also strongly associated with periodontal disease^9–11^, typically resulting from oral immune dysfunction, chronic inflammation and destruction of the periodontal tissue^12^. New studies have illustrated that CS exposure augments biofilm formation in several periodontal pathogens *in vitro*^13^, thus providing a molecular explanation for the observed clinical features.

The mechanisms behind increased susceptibility to infections as a result of cigarette smoking is not fully understood, but past studies have largely attributed this to host immune dysregulation^14–16^. CS can impair normal neutrophil cell function, decreasing phagocytic killing^17^ and chemotaxis^18^, and induce overproduction of pro-inflammatory mediators, reactive oxygen species and enzymes, resulting in extensive cellular damage and immune dysfunction^19^. Additionally, *in vitro* studies have shown that CS attenuates cellular responses to pathogen associated molecular patterns (PAMPs) leading to immunosuppression and chronic microbial colonisation^20^. Moreover, CS compromises the integrity of the respiratory epithelium increasing permeability and inhibiting normal mucociliary movement resulting in decreased microbial clearance^21^. The net effect of CS on normal immune homeostasis permits increased susceptibility to infection by opportunistic pathogens such as *Staphylococcus aureus*.

*S aureus* is a member of the nasal microflora in 30–60% of people^22,23^. This opportunistic pathogen possesses a sophisticated arsenal of virulence factors permitting a suite of infections ranging in severity from superficial skin infections to life-threatening pneumonia or endocarditis^24^. Treatment of *S aureus* infection has been made more complicated by the presence of antibiotic resistant lineages such as methicillin-resistant *S aureus* (MRSA)^25^. Historically, MRSA infections were limited to immunocompromised healthcare- associated patients. Presently, there is a growing concern regarding the emergence of community-acquired (CA-) MRSA, infections in otherwise healthy individuals outside the healthcare setting^26^.

Previous work has investigated the impact of CS on MRSA virulence^27–29^. These studies used primarily the USA300 strain (ST8 clone, staphylococcal cassette chromosome *mec* (SCC*mec*) IV; CA-MRSA) and illustrated that CS exposure resulted in a number of altered virulence phenotypes, namely augmented biofilm formation, decreased phagocytic killing, increased attachment and invasion of keratinocytes and increased severity in a mouse pneumonia model. However, MRSA is a clonal pathogen and the net result of numerous large epidemiological studies has highlighted a number of predominant clones that are responsible for the majority of the global prevalence of MRSA and subsequent disease burden^30–32^. Additionally, the variation of expression of virulence factors of different MRSA clones and isolates within the same clone has been documented, redefining the complex virulence regulation of *S aureus*^33–35^. Finally, conflicting epidemiologically data prevents a complete understanding of the relationship between smoking status and prevalence of *S aureus*^36–39^. Therefore, our aim was to assess the impact of CS on specific virulence phenotypes in a number of diverse genome-sequenced reference *S aureus* strains and to investigate any clone-specific heightened virulence as a result of CS exposure.

## Materials and Methods

### Bacterial strains and culture conditions

*S aureus* strains used in this study represent genome-sequenced reference strains from clonally distinct, clinically significant lineages (Table 1). All strains were routinely stored at −80 °C in 15% glycerol/broth stocks until required. *S aureus* strains were streaked onto tryptic soy agar (TSA), incubated for 18 h at 37 °C, and single colonies transferred to 2 mL of tryptic soy broth (TSB) in 12 mL plastic sterile tubes and propagated in a shaking incubator at 175 rpm for 24 h at 37 °C. 1 × 10^6^ CFU of overnight culture was used to subculture into TSB or 50% CS-TSB. For transposon mutants^40^ erythromycin (5 µg mL^−1^) was added to the growth medium in overnight cultures but omitted in subsequent subculture.

**Table 1:** List of strains used in this study.

Strain	Description	Reference
JKD6159	CA-MRSA; Type IV SCC*mec*; CC93; highly virulent in mouse model of skin infection; dominant Australian clone	^81^
MW2	CA-MRSA; Type IV SCC*mec*; CC1 (USA400); associated with skin and soft tissue infections; predominantly found in America	^82^
JE2	USA300 (CA-MRSA; Type IV SCC*mec*; CC8 (USA300); associated with skin and soft-tissue infections; dominant American clone) lacking plasmids p01 and p03, wild type strain of the NTML	^55^
EMRSA-15	HA/CA-MRSA; Type IV SCC*mec*; CC22; associated with a wide range of infections; globally disseminated, high prevalence in Europe	^83^
TW20	HA-MRSA; Type III SCC*mec*; CC239; associated with a wide range of infections; globally disseminated, high prevalence in Asia	^84^
MRSA252	HA-MRSA; Type II SCC*mec*; CC30; associate with haematogenous infection, bacteraemia, endocarditis, osteomyelitis; historically globally disseminated	^85^
SH1000	MSSA, laboratory strain, 8325-4 with a repaired *rsbU* gene; SigB positive	^86^
SH1000 *katA*::Tn	Catalase mutant of strain SH1000	^40^
SH1000 Δ*katA*Δ*ahpC*	Catalase and alkyl hydroperoxidase reductase mutant of strain SH1000	^51^
SH331	SH1000 *rex::*Tn;	^40^
SH445	SH1000 *umuC*::Tn;	^40^
SH805	SH1000 *recA*::Tn;	^40^
SH1012	SH1000 *rexB*::Tn;	^40^
SH1866	SH1000 *dinB*::Tn;	^40^
SCV13	SH1000 derived Gen^R^ SCV with auxotrophy for menadione isolated following growth in 50% CS	This study
Newman (ANG112)	MSSA, laboratory strain isolated from human infection	^87^
Newman Δ*tagO* (ANG260)	Wall teichoic acid mutant of strain Newman	^88^
NE95	JE2 Tn::*agrB*; accessory gene regulator B mutant	^55^
NE186	JE2 Tn::*fnbA*; fibronectin binding protein A mutant	^55^
NE1354	JE2 Tn::*hla*; alpha haemolysin mutant	^55^
NE1787	JE2 Tn::*srtA*; sortase A mutant	^55^

Abbreviations: CA-MRSA, community-associated MRSA; CC, clonal complex; NTML, Nebraska Transposon Mutant Library; SCC*mec*, Staphylococcal cassette chromosome *mec*.

### Cigarette smoke extraction

Cigarette smoke (CS) extraction protocol was optimised based on previous extraction procedures investigating the effect of CS on both eukaryotic and bacterial cell physiology^27,28,41^. A 60-mL syringe (Becton Dickinson) fitted with a polycarbonate stopcock 4-way male luer lock (Cole-Parmer), was filled with 10 mL TSB, and connected to one Marlborough cigarette (9–10 mg Tar; 0.7–0.8 mg nicotine). 50 mL of cigarette smoke was aspirated over 5 s, agitated for 15 s and slowly expelled. This process was repeated 8 times (to approximately 1 cm remaining of cigarette). The absorbance (600 _nm_) of each batch was documented and normalised to 0.35 to ensure consistency between experiments. This 10 mL CS TSB medium (CS-TSB) was defined as 100% CS-TSB. The CS-TSB broth was filtered through a 0.22-µm membrane (Millipore, PVDF low-protein binding membrane) and used immediately. Nicotine and acrolein (Sigma) were added to *S aureus* cultures grown in TSB at various concentrations to assess their impact on SCV emergence.

### *S aureus* growth analysis in TSB and CS-TSB

24 h cultures of *S aureus* strains were normalised to OD_600nm_ = 1, diluted 1:10 in TSB and 2 µL (1 × 10^5^ CFU) was used to inoculate 200 µL of TSB or 25%, 50% or 75% CS-TSB medium in a 96 well Costar plate. OD_600nm_ readings were taken every 15 min for 18 h using a Varioskan flash multimode reader (Thermo Scientific). Growth curves are graphed as the median of two independent experiments done in triplicate.

### Biofilm formation

Semi-quantitative measurements of biofilm formation on 96-well, round-bottom, polystyrene plates (Costar) was determined based on the classical, crystal violet method of Ziebuhr *et al*.^42^. 18 h bacterial cultures grown in TSB were diluted 1:40 into 100 µL TSB containing 0.5% glucose with varying percentages of CS TSB media (0–50%). Perimeter wells of the 96-well plate were filled with sterile H_2_O and plates were placed in a separate plastic container inside a 37 °C incubator and grown for 24 h under static conditions. Following 24 h growth, plates were washed vigorously five times in PBS, dried and stained with 150 μL of 1% crystal violet for 30 min at room temperature. Following five washes of PBS, wells were re-suspended in 200 μL of 7% acetic acid, and optical density at 595 nm was measured using a Varioskan flash multimode reader. Experiments were done using three technical repeats from four independent experiments.

### Vesicle Lysis Test – Agr activity

Strains were grown as described in culture conditions. Culture supernatants were centrifuged for 10 min at 20,000 × *g* and stored at −20 °C until further use. Phospholipid vesicles were prepared and the Vesicle Lysis Test to determine Agr activity was used as described previously^43^. Briefly, harvested cell-free supernatant were incubated in a 1:1 ratio with purified vesicles for 30 min. Fluorescent intensity was measured at excitation and emission wavelengths of 485 and 520 nm respectively using a Varioskan flash multimode reader. Positive and negative controls used consisted of vesicles with 0.1% triton X-100 and HEPES buffer respectively. Normalised fluorescence was achieved using the equation (Ft–F0)/(Fm/F0) where Ft is the average fluorescence value at a specific time point, F0 is the minimum and Fm is the maximum fluorescence value in that particular experiment as described previously^43^. Experiments were done in triplicate and repeated three times.

### Bronchial epithelial cell culture and cytotoxicity assay

Culture supernatant was prepared according to methods for Agr activity analysis. Type II-like bronchial epithelial cell line A549 (ATCC CCL-185) was cultured in Roswell Park Memorial Institute (RPMI; Biowest) medium with L-glutamine, supplemented with foetal bovine serum (FBS; 10%), 1% HEPES, 1% antibiotic-antimycotic solution and 0.005 M β-mercaptoethanol (Complete Medium (CM)) at 37 °C and 5% CO_2_. Epithelial cells were routinely cultured in T75 flasks (Delta Surface, Nunc) to approximately 90% confluency, liberated with trypsin-EDTA, and resuspended in CM. Cells were prepared at 5 × 10^5^ cells mL^−1^ and 75 µL were added per well. 75 µL of culture supernatant (diluted to 50% using TSB) was added, mixed and incubated for 20 min at 37 °C. Cytotoxicity was determined by the by release of lactate dehydrogenase (LDH) using the CytoTox 96 non-reactive cytotoxicity as per manufacturers’ instructions (Promega). Cytotoxicity assays were done in duplicate and repeated three times. To investigate the reversible nature of CS-mediated down regulation of toxicity, JE2 was grown for 16 h in either 0% or 50% CS-TSB in duplicate, supernatant harvested and cytotoxicity measured as above. Stationary phase cell pellets were washed three times in PBS and either resuspended in 0% CS-TSB or 50% CS-TSB and grown for 16 h and cytotoxicity measured at different time points.

### A549 cell invasion and persistence assay

Cells were added at approximately 2.5 × 10^5^ cells per well and incubated at 37 °C and 5% for 18 h, where cells were greater than 95% confluent. For invasion assay, wells were washed three times in PBS and 495 µL of media lacking antibiotic-antimycotic (CM-) was added 2 h prior to invasion. Bacterial strains grown in TSB for 18 h were sub-cultured 1:100 on the day of the experiment in either TSB or 50% CS-TSB, and grown to mid-exponential phase of growth (optical density of 0.5–0.6 OD_600nm_)). Bacterial cells were washed twice in PBS and normalised to OD_600_ of 0.3. 5 µL of washed bacteria were added to wells in duplicate and allowed to invade for 1 h. Bacterial suspensions were removed, wells washed five times in PBS and replaced with media containing 200 µg mL^−1^ gentamycin and 20 µg mL^−1^ lysostaphin for 1 h. In experiments using TW20, 100 µg mL^−1^ lysostaphin was used. For 2 h invasion, wells were washed five times in PBS, cells lysed with 0.2% triton X-100 and CFU enumerated through serial dilution and plating onto TSA plates and incubated at 37 °C for 24 h. For 24 h invasion, wells were left for a further 23 h in gentamycin/lysostaphin media and invasion enumerated as above for 2 h invasion. Invasion was calculated as the percentage of surviving cells from t (0) to t (2) or t (24). Invasion experiments were done in duplicate and repeated three times.

### Alpha haemolysin detection

Culture conditions and supernatant harvesting were performed as per Agr activity analysis. Supernatant proteins were precipitated using trichloroacetic acid, subjected to SDS-PAGE and separated proteins blotted onto nitrocellulose membrane as described previously^44^. Membranes were incubated with rabbit anti-staphylococcal alpha toxin antibody (1:5000 dilution; Sigma) and primary antibodies detected with horseradish peroxidase-coupled protein G (1:1000; Invitrogen. Immunoblost were developed by using the Opti-4CN detection kit (Bio-Rad).

### Small colony variant formation

Small colony variant (SCV) formation was examined as described by Edwards *et al*.^45^. Briefly, CFU counts were determined following growth on TSA with or without gentamicin (2 µg mL^−1^). SCVs are gentamycin resistant due to their low membrane potential and consequently lack of aminoglycoside uptake^46^. SCVs were defined as gentamicin-resistant (MIC > 2 µg ml^−1^) bacteria that produced small, slow-growing, non-pigmented or weakly pigmented colonies on TSA. SCV stability was classified as previously described^45^. Briefly, SCV stability was scored as follows: a single SCV colony was streaked onto TSA without antibiotics and incubated for 48 h. If all colonies remained as SCV it was classed as stable, if there was a mixture of SCV and WT colonies it was scored as partial and if all colonies reverted back to WT levels it was scored as unstable. To investigate the role of reactive oxygen species (ROS) as a trigger for SCV formation, the ROS inhibitor N-acetyl-L-cysteine (NAC; Sigma) was added at a final concentration of 12.5 mM. For complementation of stable SCV (SCV13), menadione (Sigma) was added to the media at a final concentration of 1 µg ml^−1^. Results represent the mean ± standard deviation (SD) of five separate experiments.

### Mutation frequency analysis

1 × 10^6^ CFU of stationary phase cultures were used to inoculate TSB and CS-TSB (25% and 50%) grown with shaking (175 rpm) for 16 h and 37 °C. Total CFU counts were determined following plating on TSA agar. To determine the number of drug resistant bacteria, cultures were plated onto TSA containing rifampicin (100 µg mL^−1^) and enumerated following incubation at 37 °C for 24 h. The incidence of resistance was calculated by dividing the number of resistant bacteria by total number of viable bacteria. Results represent the mean ± SD of five separate experiments.

### Whole genome sequencing

DNA was extracted from wild-type SH1000 and CS-derived SCV (SCV13) using the QIAamp DNA Mini Kit (Qiagen) according to manufacturers’ instructions with a pre-lysis step involving lysostaphin (0.2 mg mL^−1^; AMBI products). Purified DNA was sequenced at the Wellcome Trust Centre for Human Genetics, Oxford, on the Illumina (San Diego California, USA) HiSeq 400 platform, with 150 base-pair, paired-end reads. We used Stampy v1.0.22^47^ to map reads to a reference genome (MRSA252, NC_002952). We used Cortex^48^ for *de novo* assembly of genomes and identification of single nucleotide polymorphisms (SNPs) and short sequence insertion or deletion (indels). Quality filters were applied as previously described^49^.

### Statistical analysis

For experiments involving a comparison of the impact of 0% or 50% CS-TSB on virulence phenotype (Biofilm (Fig. 3A), Agr activity (Fig. 3B), Cytotoxicity (Fig. 4A), invasion (Fig. 5A,B), SCV and mutational frequency (Fig. 6A,B,F,G), SCV frequency over time and SCV stability (Fig. 7A,C) a two-way ANOVA with Bonferroni’s multiple comparison test was employed. For determining the impact of specific mutants in CS-mediated biofilm augmentation (Fig. 3C), the effect on toxicity after eliminating CS exposure (Fig. 4B), SCV haemolysis (Fig. 6E) and the impact of N-acetyl-L-cysteine (NAC) on SCV emergence (Fig. 7B) one-way ANOVA with Dunnett’s multiple comparison test was used.

## Results

### CA-MRSA strains appear more resistant to reactive oxygen species (ROS) present in CS-TSB than HA-MRSA or laboratory strains

**Figure 1:**
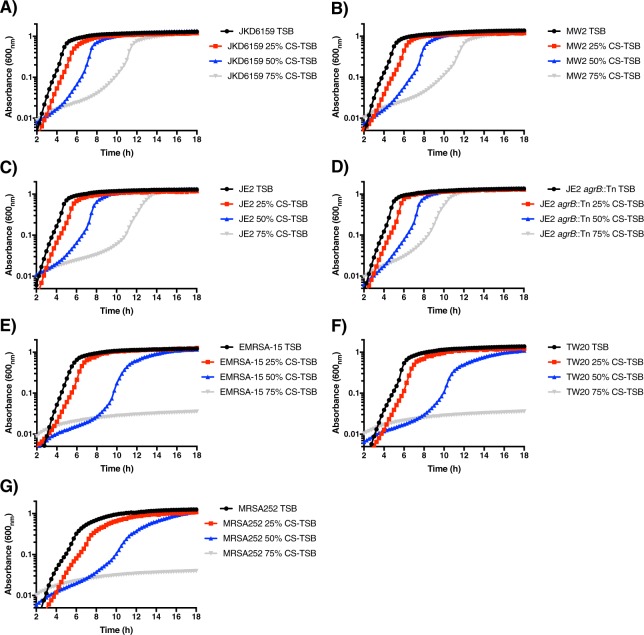
Growth curves of CA- and HA-MRSA *S aureus* strains grown in differing concentrations of CS-TSB. (**A)** JKD6159 (CA-MRSA), (**B)** MW2 (CA-MRSA), (**C)** JE2 (CA-MRSA), (**D)** JE2 *agrB*::Tn, (**E)** EMRSA-15 (HA/CA-MRSA), (**F)** TW20 (HA-MRSA), and (**G)** MRSA252 (HA-MRSA) grown with starting inocula of 1 × 10^5^ CFU, in 0% CS-TSB (black) or 25% CS-TSB (red), 50% CS-TSB (blue) or 75% CS-TSB (grey) for 18 h at 37 °C with measurements every 15 min (OD600 nm). Graphs represent the mean of two independent experiments done in triplicate.

**Figure 2:**
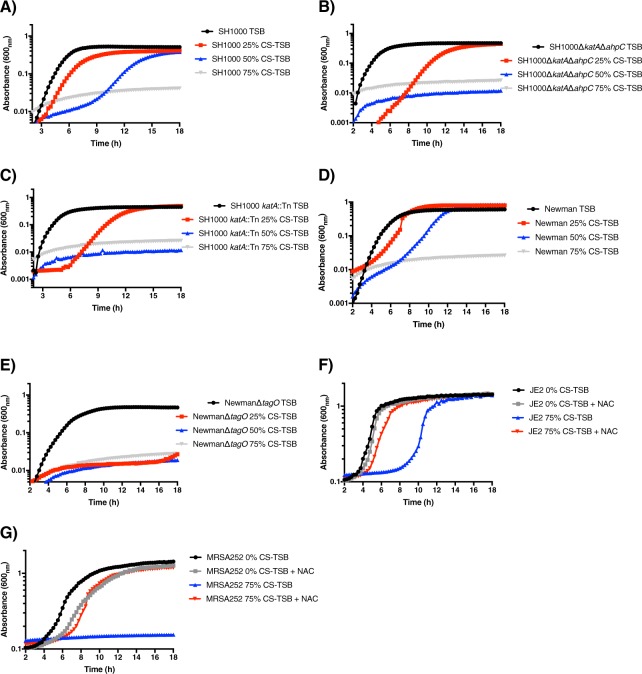
Growth dynamics of laboratory and mutant *S aureus* strains grown in differing concentrations of CS-TSB. (**A)** SH1000 (MSSA), (**B)** SH1000Δ*katA*Δ*ahpC*, (**C)** SH1000 *katA*::Tn, (**D)** Newman, (**E)** NewmanΔ*tagO*, (**F)** JE2 and (**G)** MRSA252 grown with 6.25 mM NAC in the presence or absence of 75% CS-TSB. Starting inocula of 1 × 10^5^ CFU grown for 18 h at 37 °C with measurements every 15 min (OD600 nm). Graphs represent the mean of two independent experiments done in triplicate.

Cigarette smoke contains over 4,800 compounds, some of which have growth inhibitory properties^50^. To understand at which concentration CS-TSB inhibits *S aureus* growth, we assayed a range of concentrations (0%, 25%, 50% and 75%). Interestingly we observed that *S aureus* CA-MRSA strains (JKD6159, MW2 and JE2 (USA300); Fig. 1A–C) are better able to survive and replicate within CS-TSB than HA-MRSA (EMRSA-15; CA/HA MRSA strain, TW20 and MRSA252; Fig. 1E–G) and lab strains (SH1000 and Newman; Fig. 2A,D). Inactivation of the global virulence regulator, the accessory gene regulator (Agr), had no impact on growth in CS-TSB compared to WT (Fig. 1D; strain JE2 *agrB*::Tn). *S aureus* possess detoxifying enzymes such as catalase (*katA*) and alkyl hydroperoxide reductase C (*ahpC*)^51^. In an effort to examine *S aureus* genes required to grow in CS-TSB we exposed SH1000 WT and *katA*/*ahpC* double mutant or *katA* mutant to CS-TSB and showed that *katA* is essential in permitting growth within CS-TSB (Fig. 2B,C). Cell wall teichoic acids (WTA) are abundant surface glycopolymers found in Gram-positive bacteria and play important functions in maintaining cell wall structure. Previous studies have highlighted the role of WTA in promoting intracellular survival^52^ whereby reactive oxygen species play a prominent role in microbial elimination^53^. Accordingly, we evaluated the role of WTA in protecting against CS-TSB. Interestingly, we observed that a WTA mutant (Newman*ΔtagO*; Fig. 2D,E) was hypersensitive to CS-TSB, suggesting a role of WTAs in mediating resistance to CS-TSB inhibition.

**Figure 3:**
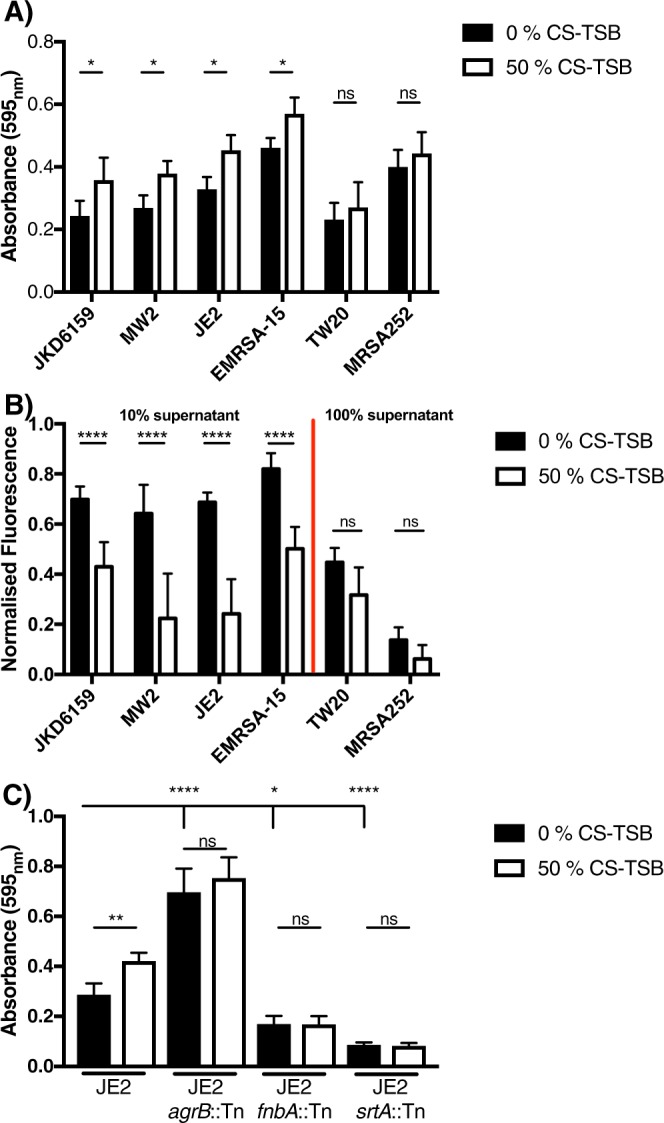
CS increases biofilm formation in a strain and Agr-dependent fashion. (**A)** Biofilm formation was evaluated using 6 genome sequenced strains following growth in 0% CS-TSB or 50% CS-TSB with 1% glucose for 24 h in polysterene 96 well plates. (**B)** Agr activity was evaluated using the vesicle lysis test, incubating self-quenched vesicles with either 10% supernatant (JKD6159, MW2, JE2 and EMRSA-15) or 100% supernatant from 18 h bacterial cultures. (**C)** Biofilm formation was evaluated in WT (JE2), JE2 *agrB*::Tn, JE2 *fnbA*::Tn and JE2 *srtA*::Tn mutants grown with and without 50% CS-TSB. Graphs represent the mean ± SD and significant differences measured using a two-way ANOVA with Bonferroni’s multiple comparison test (**A,B)** or a one-way ANOVA with Dunnett’s multiple comparison test was used (**C**) (*p < 0.05; **p < 0.01; ***p < 0.001; ****p < 0.0001).

**Figure 4:**
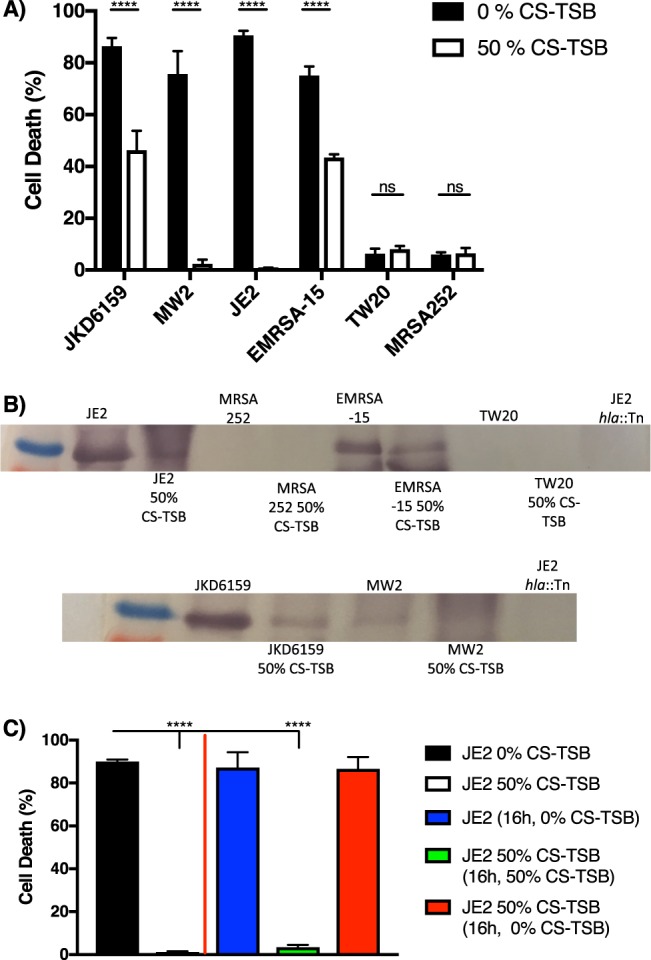
CS reduces cytolytic activity of *S aureus* in a reversible manner. (**A)** *S aureus* strains were grown in 0% CS-TSB or 50% CS-TSB for 16 h and cytolytic activity measured following incubation of bacterial supernatant with A549 cells and cell death determined by release of lactate dehydrogenase. (**B)** Alpha haemolysin detection by western blot of concentrated *S aureus* supernatant of strains either grown in the presence or absence of 50% CS-TSB. The alpha heamolysin mutant (JE2 *hla*::Tn) is used as a control. (**C)** Toxin production is rescued when CS exposure is lifted. JE2 was grown in 0% CS-TSB (black bar) or 50% CS-TSB (white bar) for 16 h and cytolytic activity of supernatants assessed. Cell pellets of 0% CS-TSB were washed and reintroduced into 0% CS-TSB and cytolytic activity assessed after 16 h (blue bar). Cell pellets of 50% CS-TSB were washed and reintroduced into either 50% CS-TSB (green bar) or 0% CS-TSB (red bar) and cytolytic activity assessed after 16 h. Graphs represent the mean ± SD and significant differences measured using a two-way ANOVA with Bonferroni’s multiple comparison test (**A**) or a one-way ANOVA with Dunnett’s multiple comparison test was used (**C**)(****p < 0.0001).

**Figure 5:**
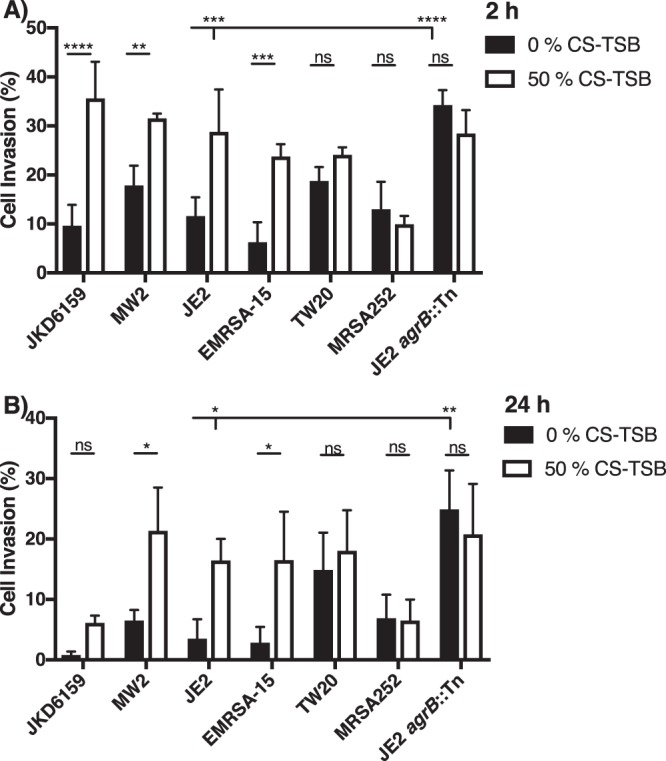
CS-mediated increased *S aureus* invasion and persistence within bronchial epithelial cells requires a functional Agr. CS promotes the invasion of *S aureus* strains harbouring a functional Agr system as observed by increased intracellular survival following (**A)** 2 h and (**B)** 24 h incubation when grown in 50% CS-TSB compared to 0% CS-TSB. Graphs represent the mean ± SD and significant differences measured using a two-way ANOVA with Bonferroni’s multiple comparison test (*p < 0.05; **p < 0.01; ***p < 0.001; ****p < 0.0001).

**Figure 6:**
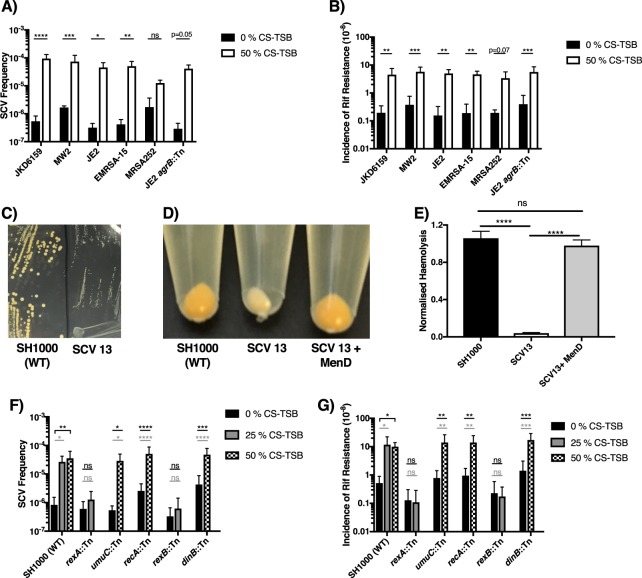
Growth in 50% CS-TSB increases the frequency of gentamicin resistant SCVs and mutation rate. *S aureus* strains were grown in the presence or absence of 50% CS-TSB for 16 h and (**A)** SCV frequency and (**B)** mutation rate were determined. (**C)** Colony morphology of WT (SH1000) and SCV isolated after growth in 50% CS-TSB for 16 h (SCV 13). Colonies were sub-cultured onto TSA containing no antibiotics for 48 h. (**D)** Lack of carotenoid pigment and (**E)** haemolytic activity of the SCV is observed following growth on TSA agar or following incubation of 24 h SCV supernatant with human red blood cells. Addition of 1 µg ml^−1^ menadione restored pigment production and haemolytic activity suggesting defects in menaquinone production. (**F**) SCV frequency and (**G**) mutation rate in WT (SH1000) and in mutants of the SOS DNA mutagenic pathways (*rexA*; *umuC*; *recA*; *rexB; dinB*) in 0%, 25% and 50% CS-TSB were determined. Graphs represent the mean ± SD and significant differences measured either by using a two-way ANOVA with Bonferroni’s multiple comparison test (**A,B,F,G**) or a one-way ANOVA with Dunnett’s multiple comparison test (**E**) (*p < 0.05; **p < 0.01; ***p < 0.001; ****p < 0.0001).

**Figure 7:**
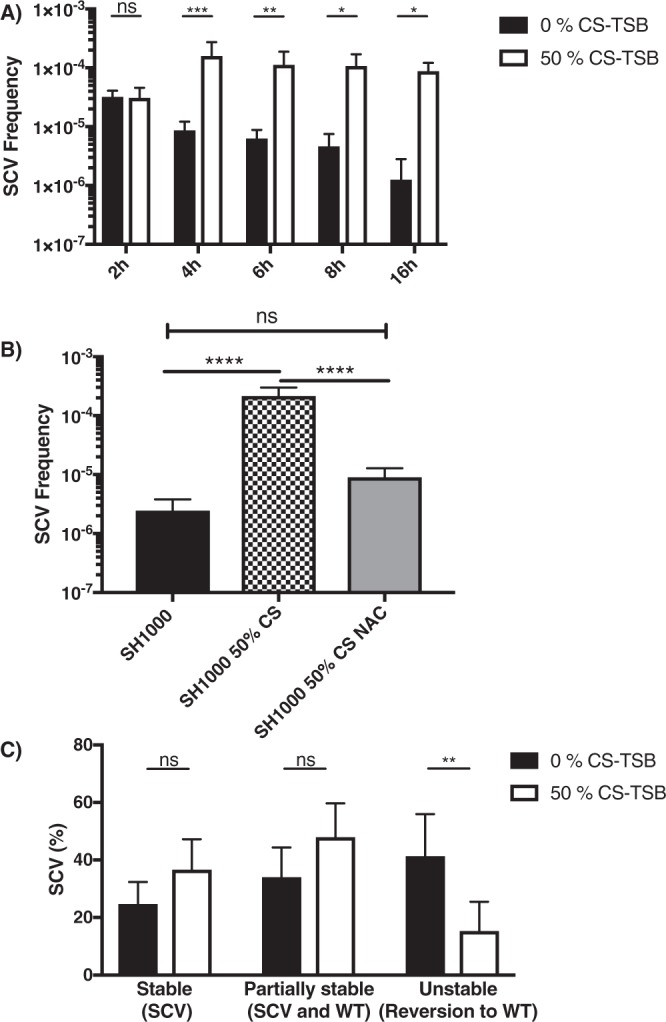
Short term CS exposure induces stable SCVs. (**A)** *S aureus* (SH1000) was incubated in 0% or 50% CS-TSB and SCV frequency was assessed at 2, 4, 6, 8 and 16 h of growth. (**B)** Treatment of 50% CS-TSB with 12.5 mM N-acetyl-L-cysteine significantly reduced the frequency of SCV. (**C**) The stability of SCVs isolated from growth in 0% CS-TSB or 50% CS-TSB was determined as outlined in Materials and Methods. Graphs represent the mean ± SD and significant differences measured either by using a two-way ANOVA with Bonferroni’s multiple comparison test (**A,C**) or a one-way ANOVA with Dunnett’s multiple comparison test (*p < 0.05; **p < 0.01; ***p < 0.001; ****p < 0.0001).

A key component of CS is reactive oxygen species (ROS), which at high concentrations are bactericidal^53^. To further confirm the role of ROS in *S aureus* growth inhibition at high concentrations of CS-TSB, we incubated CS-TSB with a ROS inhibitor, N-acetyl-L-cysteine (NAC), prior to bacterial inoculation. The addition of NAC reversed growth inhibition of both CA-MRSA (Fig. 2F, JE2) and HA-MRSA (Fig. 2G; MRSA252) strains to levels seen in 0% CS-TSB. In accordance to the results displayed in both Figs 1 and 2, we used 50% CS-TSB throughout our experiments as it was the highest concentration of CS with the lowest growth inhibitory properties and mirrored conditions used previously to evaluate CS-mediated modulation of *S aureus* virulence^27,28^.

### CS promotes biofilm formation in a functional Agr-dependent fashion

Using 6 clonally distinct strains (Table 1) we observed a strain dependent increase in biofilm formation following growth in 50% CS-TSB (Fig. 3A). Of the 6 strains sampled, two strains, MRSA252 and TW20, showed no significant change in biofilm biomass, whereas JKD6159, MW2, JE2 and EMRSA-15 and all showed significantly increased biofilm formation, underpinning that distinct *S aureus* strains respond differently to CS-TSB.

Previous work by Kulkarni *et al*. illustrated a decrease in *rnaIII expression*, the effector molecule of the Agr system, a regulatory network central to virulence gene expression in *S aureus*, following exposure to CS^27,54^. We investigated the Agr activity of the above strains in the presence and absence of 50% CS-TSB (Fig. 3B). *S aureus* strains that formed significantly more biofilm in the presence of 50% CS-TSB had higher Agr activity under normal growth conditions (0% CS-TSB) and had significantly reduced Agr activity in the presence of 50% CS-TSB (JKD6159, MW2, JE2 and EMRSA-15) (Fig. 3A,B). In contrast, strains MRSA252 and TW20 which had no significant biofilm alteration in the presence of 50% CS-TSB, had lower Agr activity which was not affected by growth in 50% CS-TSB (Fig. 3A,B). Of note MRSA252 and TW20 had significantly lower Agr activity than the other strains, highlighted by the fact that 100% supernatant was used in Agr activity analysis compared to 10% for the high Agr active group of strains (Fig. 3B).

To fully confirm the mechanism behind CS-mediated increased biofilm we tested the biofilm forming capacity of three mutants known to be important in this process, in the presence and absence of 50% CS-TSB: an Agr mutant (JE2 *agrB*::Tn) where the majority of cell surface proteins are repressed following activation of Agr^54,55^, a previously determined biofilm promoting surface protein, fibronectin binding protein A^56^, FnBPA (JE2 *fnbA*::Tn) and sortase A (JE2 *srtA*::Tn), an enzyme responsible for anchoring surface proteins to the cell wall^57^. Under 0% CS-TSB conditions an increase in biofilm was observed in the Agr mutant whereas a decrease was seen in mutants with impaired surface protein(s). Under 50% CS-TSB conditions we only saw an increase in WT strains, and not in mutants, highlighting the importance of an active, functional Agr system and expression of surface proteins, particularly FnBPA, in the augmentation of biofilm formation in *S aureus* in response to CS exposure (Fig. 3C).

### CS reduces toxin production in a reversible manner

*S aureus* secretes a multitude of cytolytic toxins that interact with and lyse diverse host cell types^58^, where Agr plays a key role in upregulating toxin production in a growth-phase dependent fashion^54^. To examine the impact of CS on toxin expression we assessed cytolytic activity of *S aureus* supernatant following growth in the presence or absence of 50% CS-TSB using a bronchial alveolar epithelial (A549) cell line (Fig. 4A). Unsurprisingly, strains with high Agr activity were most toxic, (JKD6159, MW2, JE2 and EMRSA-15) whereas those with low Agr activity had very low levels of toxicity (MRSA252 and TW20).

In the highly cytolytic strains, growth in 50% CS-TSB led to a significant decrease in toxin production in accordance with CS-mediated downregulation of Agr. We also observed significant differences in the degree of reduction of toxin production following growth in CS-TSB, whereby cytolytic activity was highly attenuated in JE2 and MW2 compared to EMRSA-15 and JKD6159 (Fig. 4A), reinforcing strain-dependent differences in *S aureus* response to CS exposure. CS exposure had no effect on toxicity in low cytolytic strains (MRSA252 and TW20). To confirm down regulation of toxin production following growth in 50% CS-TSB, we measured the expression of the major virulence factor, alpha haemolysin, by western blotting. Consistent with our toxicity results, highly cytolytic strains exhibited a marked reduction in alpha- haemolysin expression (Fig. 4B). Note that neither TW20 nor MRSA252 strains produced detectable alpha-haemolysin.

Next, we wanted to investigate whether this reduction in toxicity was a stable phenotype, resulting from mutation following CS exposure, or a reversible characteristic following removal of CS. Here JE2 was grown in the presence and absence of 50% CS-TSB for 16 h, supernatants harvested and cytolytic activity assessed (Fig. 4C). Cell pellets from both 0% and 50% CS-TSB were washed thoroughly in PBS to remove residual CS components. 0% CS-TSB cell pellet was resuspended in 0% CS-TSB whereas the cell pellet isolated from 50% CS-TSB was resuspended in either 0% CS-TSB or 50% CS-TSB. Cultures were then incubated and toxin production assessed at time 2, 4, 8, and 16 h post inoculation. No toxicity to either vesicles or bronchial epithelial cells was observed from supernatants isolated from time points 2–8 h (data not shown) under any conditions. However, cell pellets from 0% or 50% CS-TSB reintroduced to 0% CS-TSB resulted in toxin expression following 16 h growth, whereas significant attenuation in toxicity remained following growth in 50% CS-TSB (Fig. 4C), suggesting that CS attenuates toxin production in a reversible manner when the oxidative stress is lifted.

### CS-mediated increased *S aureus* invasion and short-term persistence within bronchial epithelial cells requires a functional Agr

*S aureus* has the capacity to invade various cell types which affords the bacterium protection from immune surveillance and clearance from the majority of antibiotic therapies^59^. Previous studies concluded that Agr mutants have a higher invasive potential owing to their inability to down regulate surface proteins^60^. Given the effect of CS on Agr we wanted to investigate whether this effect translated into an increased invasive phenotype. Four strains which expressed high Agr activity when grown in 0% CS-TSB, (JKD6159, MW2, JE2 and EMRSA-15) all exhibited an increased invasive capacity as a result of growth in 50% CS-TSB following 2 h (Fig. 5A) and 24 h (Fig. 5B) post-infection as illustrated by higher intracellular colony forming units. Strain TW20 and MRSA252 showed no significance difference in invasion or persistence following growth in 50% CS-TSB. The Agr mutant (JE2 *agrB*::Tn) resulted in a 4-fold increase in invasion compared to the wild-type strain JE2. Exposure of the Agr mutant to CS resulted in no significant difference in initial invasion or following 24 h incubation compared to normal conditions, highlighting the importance of a functional Agr system in mediating CS-induced increased invasion.

### Growth of *S aureus* in 50% CS-TSB increases both the frequency of gentamycin-resistant SCV and mutation frequency

Small colony variants (SCVs) of *S aureus* are a slow-growing, antibiotic-resistant, morphologically and phenotypically distinct sub-populations, central in long-term persistence within the host^46^. The exact triggers for SCV emergence are not fully understood but stressful conditions including antibiotic pressure^61^, nutrient limitation^62^ and chemical stresses generated by the host^40^ are known to induce SCV formation. Accordingly, we investigated whether CS exposure could trigger the emergence of SCVs. Following 16 h growth in 50% CS-TSB, all tested *S aureus* isolates bar MRSA252 exhibited a statistically significant increase in the population frequency of gentamycin-resistant SCVs (Fig. 6A). To explore the basis for this, we determined the impact of CS exposure on the mutation frequency (Fig. 6B). Our results illustrate a significant or border significant increase in the frequency of mutations in several different strains, confirming the mutagenic effect of CS exposure in *S aureus*. To characterise CS-induced SCVs, a randomly chosen isolate (SCV 13) was investigated for carotenoid pigment production and haemolysis (Fig. 6C–E). SCV13 had lower carotenoid pigment and haemolytic activity compared to WT strain SH1000, a hallmark of *S aureus* SCVs^46^. Supplementation of SCV13 with menadione (1 µg ml^−1^) restored WT levels of carotenoid pigment and haemolytic activity, suggesting a mutation in the menadione biosynthetic genes, a pathway frequently defective in SCVs^46,63^. Whole genome sequencing of WT SH1000 and SCV13 confirmed a base deletion in the isochorismate synthase (*menF*) gene in SCV13, resulting in a frameshift and predicted premature stop codon at codon 144 of 403, abolishing the first step of menadione biosynthesis. Two single nucleotide polymorphisms (SNPs) were also discovered, both predicted to be non-synonymous SNPs in membrane transport proteins (SAUSA300_0852; *mnhD* and SAUSA300_1642; *aapA*). However, no role for these genes has been observed in SCV formation.

### CS induced SCVs occur through DNA recombinational repair via the RexAB pathway

Recently, work by Painter *et al*. showed that exposure to sub-lethal concentrations of hydrogen peroxide induced SCV formation in *S aureus* via the SOS mutagenic DNA-repair pathway^40^. We therefore hypothesized that CS may activate the SOS response, resulting in elevated mutation rate and SCV emergence. To test this, we evaluated the SCV and mutational frequency in the presence and absence of CS-TSB in a set of mutants with transposon insertions in genes required for SOS induction and DNA repair in *S aureus* (Table 1; Fig. 6F,G); including recombinase A (SH1000*recA*::Tn), trans-lesion DNA polymerases IV and V (SH1000*dinB*::Tn and SH1000*umuC*::Tn respectively) and *rexAB*, genes involved in DNA recombinational repair (SH1000*rexA*::Tn and SH1000*rexB*::Tn respectively). We identified the RexAB pathway as essential in mediating the emergence of SCV following CS exposure. Disruption of the *rexAB* genes prevented emergence of both gentamicin SCVs and rifampicin mutants following growth in 25% CS-TSB compared to the wild-type SH1000 strain. In this case we compared the *rexAB* mutants and wildtype using 25% CS-TSB, as 50% CS-TSB resulted in total inhibition of growth of the *rexAB* mutants (Supplementary Fig. 1), suggesting that CS exposure results in double stranded DNA breaks in *S aureus* which is bactericidal in mutants of DNA recombinational repair.

### Short-term CS exposure induces stable SCVs

Exposure times as little as 4 hours resulted in a significant increase in SCV frequency following growth in 50% CS-TSB, which continued at 6, 8 and 16 h of growth (Fig. 7A). In an effort to understand which class of chemicals was responsible SCV emergence we incubated 50% CS-TSB with NAC for 16 h and compared SCV frequency values obtained from growth in TSB NAC and 50% CS-TSB (Fig. 7B). Following NAC treatment of 50% CS-TSB, we observed a significant decrease in SCV frequency compared to growth in 50% CS-TSB. Additionally, we observed no significant difference in SCV frequency when grown in TSB NAC and 50% CS-TSB NAC, highlighting that reactive oxygen species within CS constituent a major trigger for SCV emergence. The effects of purified nicotine and acrolein, key toxic components in tobacco and cigarette smoke, on SCV emergence were also assessed however no significant difference in SCV frequency was observed (Supplementary Fig. 2).

SCVs are known to revert back to WT levels quite rapidly following removal of stressful conditions^45^. Therefore, we examined the stability of SCVs established with or without growth in 50% CS-TSB as previously described^45^. Significantly more colonies reverted back to wild-type when grown in TSB compared to growth in 50% CS-TSB (Fig. 7C), illustrating that CS exposure results in significantly more stable SCVs.

## Discussion

Smoking represents a major global health issue leading to enhanced risk of cancer and immune dysfunction. What is not clear is how smoking affects members of the human microbiome, in particular opportunistic pathogens such as *S aureus*. Here we detail the strain-dependent adaptation and virulence modulation of *S aureus* to CS exposure.

Studies investigating the risk of CS on *S aureus* colonisation are conflicting, with reports suggesting that smoking can be considered a risk factor^36,37,64,65^, protective factor^38,39,66^ or having no association^67,68^ with *S aureus* prevalence. Using a panel of genetically distinct *S aureus* strains our data suggests that CA-MRSA strains were better able proliferate at high CS-TSB concentrations compared to HA-MRSA or common lab strains. Additionally, bacterial factors such as catalase, WTA and a functional recombinational repair pathway were shown to be vital for *S aureus* growth in CS-TSB. Furthermore, inhibition of reactive oxygen species permitted growth of susceptible *S aureus* strains to levels seen in the absence of CS. Our results suggest that both the degree of CS exposure and the genetic background of the colonising strain are important determinants in whether CS promotes or protects against *S aureus* colonisation. Interestingly, recent work showed that CA-MRSA strains produce significantly more WTA content than HA-MRSA or laboratory strains^69^, suggesting that WTA expression may be an important factor in mediating resistance to CS-TSB and an important prerequisite for MRSA colonisation in smokers.

The Agr system is important in controlling the temporal expression of surface proteins and toxins and is central in the ability of *S aureus* to form biofilms and invade and persist within host cells^54^. Kulkarni *et al*. have showed that Agr is affected at the transcriptional level, whereby *agrC* transcripts were significantly down-regulated following CS exposure^27,29^. Here, by using Agr mutants and experimentally determined high and low Agr active strains we confirm the importance of a functional Agr system in mediating enhanced biofilm formation and host cell invasiveness as a result of CS exposure. We illustrate that Agr mutants and isolates with low Agr activity remain unchanged in biofilm formation and invasiveness following exposure to CS, a feature that has not been previously recognised. These results are noteworthy considering that Agr dysfunctional strains represent a significant proportion of clinical isolates^70^.

*S aureus* toxicity is central to virulence in many different animal models of infection^71^. Our results show that *S aureus* isolates with high Agr activity have reduced toxicity following CS exposure due to a reduced expression of Agr. Moreover, we found that the degree of reduction in cytolytic activity varies with different strains, whereby strains JE2 and MW2 were highly attenuated following growth in 50% CS-TSB compared to strains JKD6159 and EMRSA-15, highlighting the strain dependent variation of virulence modulation imposed by CS exposure. Within the DNA-binding domain of AgrA, the transcriptional activator of the Agr system, exists two cysteine residues that, under oxidative conditions, form an intracellular disulphide bridge which results in the dissociation of AgrA from DNA, inhibiting *agr* activity^72^. In line with these observations we illustrate that *S aureus* toxicity is affected following CS exposure due to down regulation of Agr. Additionally, we show that this decrease in toxicity is not a permanent feature and if the oxidative stress is lifted toxicity can return to normal levels. Although toxicity is a significant feature in the virulence arsenal of *S aureus*, mounting evidence suggests the role of toxins to be disease-specific^33,73^, and in fact limiting toxin expression may be beneficial for progression of invasive diseases such as bacteraemia and may also contribute to intracellular persistence^60,74^.

*S aureus* can survive harsh conditions, either generated by the host (reactive oxygen species expressed by host immune cells) or by therapeutic antibiotic intervention, via several distinct mechanisms^75^, one of which is through the formation of SCVs^40,62,76^. Here, we report that CS triggers the emergence of SCVs in a cohort of genotypically diverse and clinically relevant *S aureus* isolates. Furthermore, we highlight the mutagenic effect which CS imposes on *S aureus* and confirm by mutational analysis the role of the DNA recombinational repair pathway, RexAB, in mediating the accelerated mutation rate and subsequent increased SCV frequency. The RexAB pathway is activated following double stranded (ds) DNA breaks and induction of this pathway has the capacity to accelerate the mutation rate through the formation of mutational hotspots^77^. As a result, we observed increased frequency of rifampicin resistance following CS exposure and speculate that resistance to other antibiotics, where resistance is determined by target site mutations such as for fluoroquinolones, is also likely to be affected. The exact effect of smoking on bacterial mutation rate and SCV emergence *in vivo* is unclear. Interestingly, recent work has shown that smoking induces the formation of ds DNA breaks in human cells *in vivo*^78^, providing credibility for our hypothesis that CS may induce similar DNA damage in *S aureus* and other bacteria habiting the nasopharynx, accelerating mutation rate, SCV emergence and antibiotic resistance.

We acknowledge that brief exposure to cigarette smoke *in vitro* is different to inhaled smoke over prolonged time periods. Nevertheless, previous groups have highlighted that *in vitro* CS exposure modulates virulence gene expression in *S aureus* and biofilm formation in respiratory and periodontal pathogens^6,8,13^. Taking these studies together we hypothesise that stressful conditions imposed by CS induces cellular stress responses in both host and microbial cells permitting adaptation to harsh conditions with the net effect of increasing virulence and/or potential for infection. Additionally, in certain diseases where smoking is a predominant risk factor in recalcitrant infections such as chronic rhinosinusitis (CRS), the role *S aureus* SCVs in mediating this recurrence has been suggested. Two studies by Clement *et al*.^79^ and Tan *et al*.^80^, illustrated intracellular reservoirs containing *S aureus* SCVs derived from sinonasal specimens of CRS patients suggesting that the increased invasiveness and persistence linked to SCVs may play an important role in the recurrent nature of this pathology. It is tempting to speculate that smoking may play multiple roles in promoting CRS; disruption of mucociliary clearance mechanisms of sinonasal epithelium, downregulation of local innate and adaptive immune responses and induction of SCVs, all favouring *S aureus* colonisation and subsequent re-infection.

Taken together our results highlight that CS redirects *S aureus* to a virulence profile associated with persistent infection; that is increased biofilm formation, reduced toxicity, increased invasiveness, intracellular persistence and SCV formation. However, we also demonstrate that specific virulence phenotypes vary among *S aureus* strains and care must be taken when extrapolating data on the effects of environmental stress and *in vitro* virulence. As with previous work illustrating the link between CS and heightened bacterial virulence, we hope that our data will provide a further incentive for people not to smoke and for current smokers to quit.

## Supplementary information

Supplementary Figures 1-2
