# Supplementary material for: Cigarette smoke exposure redirects Staphylococcus aureus to a virulence profile associated with persistent infection
Source: Sci Rep. 2019 Jul 25;9:10798. doi: 10.1038/s41598-019-47258-6 (PMC6658544; doi:10.1038/s41598-019-47258-6)
Supplement: Supplementary file 1 — Supplementary Figures 1-2 [file 41598_2019_47258_MOESM1_ESM.pdf]

Supplementary Figures

Cigarette smoke exposure redirects *Staphylococcus aureus* to a virulence profile associated with persistent infection

Alicia Lacoma [a, b], Andrew M Edwards [c], Bernadette C Young [d], José Domínguez [a, b], Cristina Prat [a, b], **Maisem Laabei [a, e #]**

Servei de Microbiologia, Hospital Universitari Germans Trias i Pujol, Institut d'Investigació Germans Trias i Pujol, Universitat Autònoma de Barcelona, Badalona, Spain [a]; CIBER Enfermedades Respiratorias, Badalona Spain [b]; MRC Centre for Molecular Bacteriology and Infection, Imperial College London, London, United Kingdom [c]; Nuffield Department of Medicine, Experimental Medicine Division, University of Oxford, Oxford, United Kingdom [d]; Department of Biology and Biochemistry, University of Bath, Bath, United Kingdom [e]

Running Head: Cigarette smoke alters *S aureus* virulence

# Address correspondence to Maisem Laabei ml418@bath.ac.uk

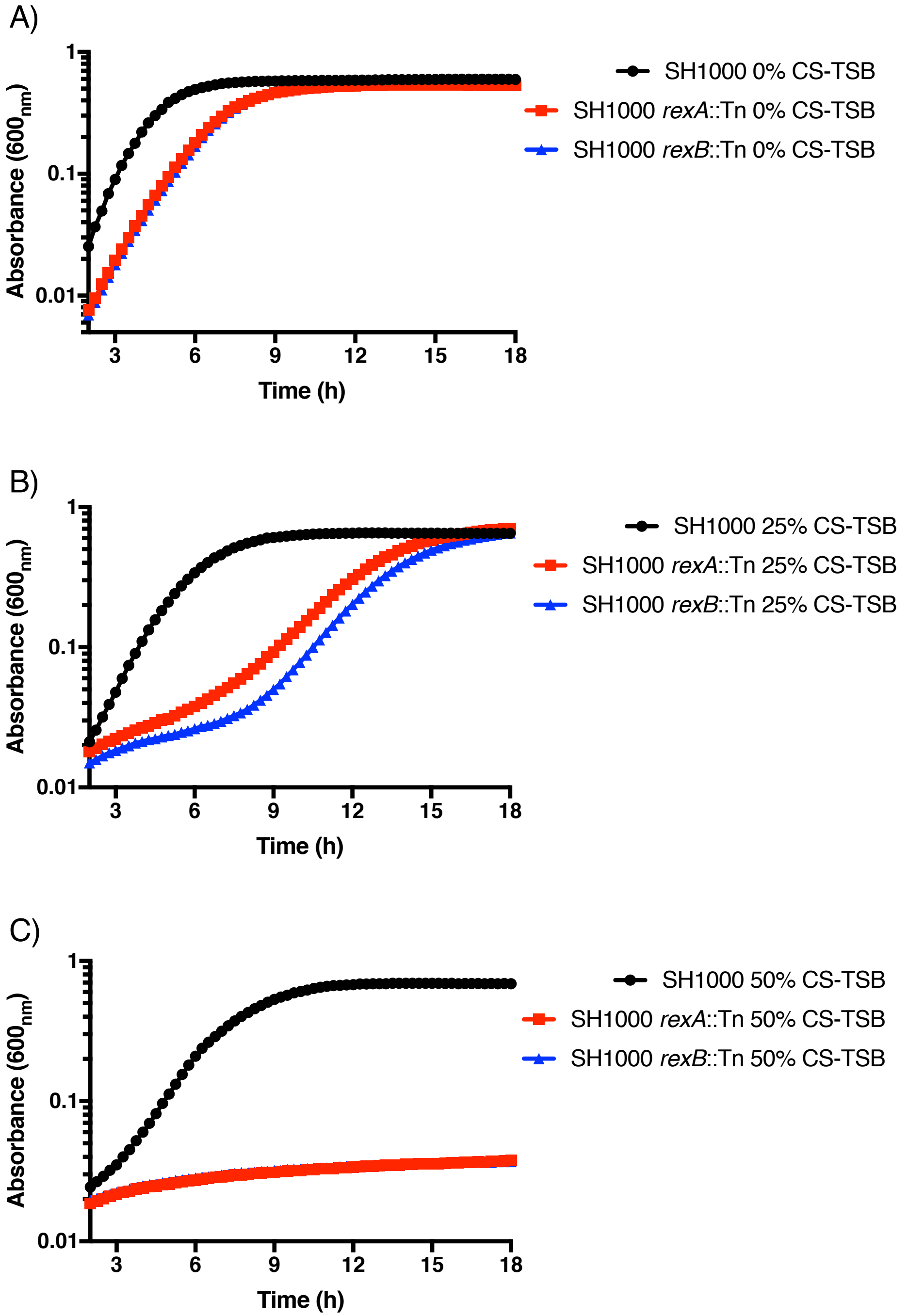

Supplementary Figure 1: Growth curves of SH1000 and *rexAB* mutants. Growth of SH1000 (black), *rexA*::Tn (red) and *rexB*::Tn (Blue) in **A)** 0% CS-TSB, **B)** 25% CS-TSB and **C)** 50% CS-TSB from a starting inoculum of 1 x 10^5 CFU for 18 h at 37 °C with measurements every 15 min (OD600nm). Graphs represent the mean of two independent experiments done in triplicate.

A)

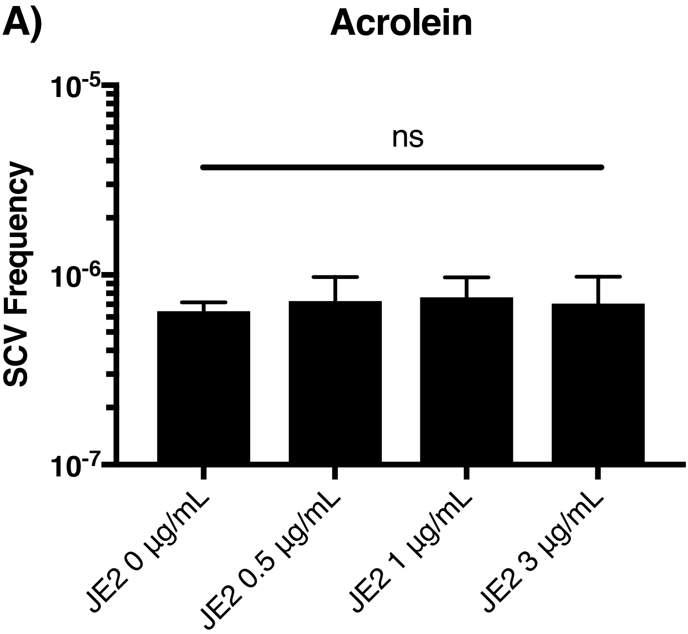

B)

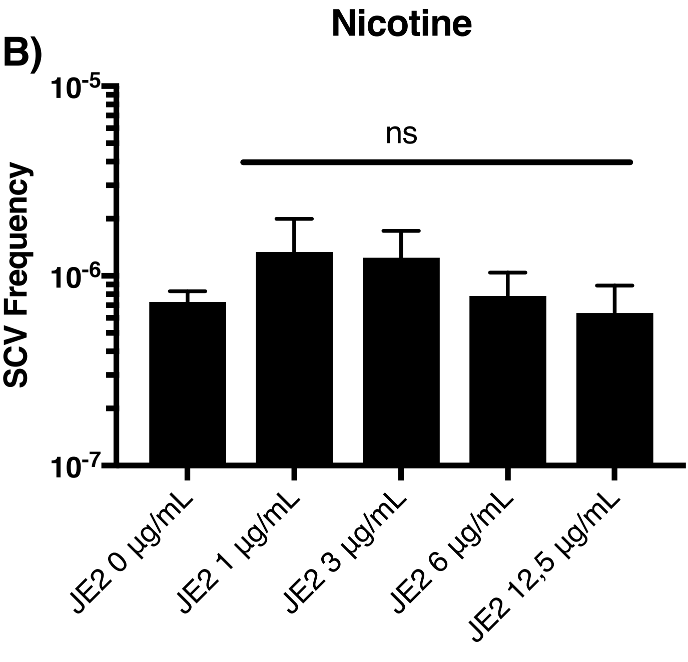

Supplementary Figure 2: Acrolein and nicotine do not induce SCV formation. S aureus strain JE2 was grown in 0% CS-TSB supplemented with increasing concetrations of **A)** acrolein and **B)** nicotine for 16 h and the SCV frequency was determined. Graphs represent the mean ± SD and significant differences measured by a one-way ANOVA with Dunnett's multiple comparison test.
